# Experimental Infection of Calves by Two Genetically-Distinct Strains of Rift Valley Fever Virus

**DOI:** 10.3390/v8050145

**Published:** 2016-05-23

**Authors:** William C. Wilson, A. Sally Davis, Natasha N. Gaudreault, Bonto Faburay, Jessie D. Trujillo, Vinay Shivanna, Sun Young Sunwoo, Aaron Balogh, Abaineh Endalew, Wenjun Ma, Barbara S. Drolet, Mark G. Ruder, Igor Morozov, D. Scott McVey, Juergen A. Richt

**Affiliations:** 1 United States Department of Agriculture, Agricultural Research Service, Arthropod Borne Animal Disease Research Unit, 1515 College Ave., Manhattan, KS 66502, USA; nng5757@vet.k-state.edu (N.N.G.); Barbara.Drolet@ars.usda.gov (B.S.D.); mgruder@uga.edu (M.G.R.); Scott.McVey@ars.usda.gov (D.S.M.); 2 Department of Diagnostic Medicine/Pathobiology, College of Veterinary Medicine, Kansas State University, Manhattan, KS 66502, USA; asally@vet.k-state.edu (A.S.D.); bfaburay@vet.k-state.edu (B.F.); jdtrujillo@vet.k-state.edu (J.D.T.); vinays@vet.k-state.edu (V.S.); sunwoo@ksu.edu (S.Y.S.); balogh@vet.k-state.edu (A.B.); adendale@vet.k-state.edu (A.E.); wma@vet.k-state.edu (W.M.); imorozov@vet.k-state.edu (I.M.); 3 Department of Diagnostic Medicine/Pathobiology, College of Veterinary Medicine, Kansas State University, Manhattan, KS 66502, USA; 4 Southeastern Cooperative Wildlife Disease Study, College of Veterinary Medicine, University of Georgia, Athens, GA 30602, USA

**Keywords:** Challenge model, Rift Valley fever, Rift Valley fever virus, Cattle, pathogenicity

## Abstract

Recent outbreaks of Rift Valley fever in ruminant livestock, characterized by mass abortion and high mortality rates in neonates, have raised international interest in improving vaccine control strategies. Previously, we developed a reliable challenge model for sheep that improves the evaluation of existing and novel vaccines in sheep. This sheep model demonstrated differences in the pathogenesis of Rift Valley fever virus (RVFV) infection between two genetically-distinct wild-type strains of the virus, Saudi Arabia 2001 (SA01) and Kenya 2006 (Ken06). Here, we evaluated the pathogenicity of these two RVFV strains in mixed breed beef calves. There was a transient increase in rectal temperatures with both virus strains, but this clinical sign was less consistent than previously reported with sheep. Three of the five Ken06-infected animals had an early-onset viremia, one day post-infection (dpi), with viremia lasting at least three days. The same number of SA01-infected animals developed viremia at 2 dpi, but it only persisted through 3 dpi in one animal. The average virus titer for the SA01-infected calves was 1.6 logs less than for the Ken06-infected calves. Calves, inoculated with either strain, seroconverted by 5 dpi and showed time-dependent increases in their virus-neutralizing antibody titers. Consistent with the results obtained in the previous sheep study, elevated liver enzyme levels, more severe liver pathology and higher virus titers occurred with the Ken06 strain as compared to the SA01 strain. These results demonstrate the establishment of a virulent challenge model for vaccine evaluation in calves.

## 1. Introduction

The mosquito-borne Rift Valley fever virus (RVFV) is a zoonotic pathogen within the genus *Phlebovirus* family *Bunyaviridae*. The virus has a tripartite, single-stranded negative-sense RNA genome. The large segment (L) encodes the RNA-dependent RNA polymerase. The medium segment (M) encodes two major envelope glycoproteins, Gn and Gc, and a non-structural protein, NSm. The S-segment utilizes an ambisense strategy to encode the nucleocapsid protein and the non-structural protein, NSs, which has been shown to be an interferon antagonist [1]. Although large outbreaks have predominantly occurred in Sub-Saharan Africa, outbreaks outside of the African continent, in the Arabian Peninsula, have raised concerns about the potential spread of the virus to Europe and the Americas [2,3]. These concerns are warranted given that North America has large, economically-important populations of susceptible animals and indigenous vector populations, experimentally shown to be competent for virus infection and transmission [4,5,6]. Typically, outbreaks of disease are characterized by abortion storms and high rates of mortality in young sheep, goats and cattle [7,8]. Livestock are considered to be the amplifying and/or reservoir hosts for human infections. Thus, outbreaks in livestock lead to human infections, resulting in acute febrile illness that in some cases can progress to more severe disease, including retinal vasculitis, resulting in blindness, encephalitis and fatal hepatitis with hemorrhagic fever [9]. Although reported human case fatality rates are generally low, higher fatality rates (20%–40%) have been noted [10,11].

RVFV is a Category A pathogen [12], and the potential for RVFV as a bioterrorism agent is widely recognized [13]. Therefore, the intentional or unintentional introduction of RVFV into the U.S. is both an agricultural and public health concern and requires a “One Health” approach. One primary control method is through livestock vaccination [14,15]. Killed or live attenuated vaccines are currently used in endemic countries. Historically, the commonly-used veterinary vaccine is an attenuated vaccine developed in 1949 known as the Smithburn strain [16]. A more recently introduced attenuated vaccine is Clone 13, which has a deletion in the NSs interferon antagonist gene and has been experimentally proven to be safe and efficacious [17,18]. Another attenuated vaccine that is considered to be safe is MP12 [19,20,21], which is conditionally licensed for use in the U.S. [22]. MP12 has also been evaluated for potential human use [23,24]. There are concerns with the use of attenuated vaccines due to potential teratogenicity [25] and potential reversion to virulence or reassortment with RVFV field strains. A number of novel vaccine approaches are in development [1], the most recent being an adenovirus-vectored vaccine [26]. With multiple inactivated, live attenuated and other new generation potential vaccines, there is a critical need for consistent, reliable vaccine efficacy animal models based on the target species (*i.e.*, sheep, goats or cattle). Animal models are available [27], and because abortion is a hallmark of RVFV of livestock, a common model is the pregnant ewe [28]. However, the pregnancy synchronization of ewes and the scheduling of limited high biosecurity animal space makes this model logistically difficult. Another model that has proven useful is vaccine-age (~4-month-old) sheep and goats [29]. This study involved different breeds of sheep (Suffolk cross, Rideau Arcott cross, Ile-de-France cross with Rideau Arcott) and a human-derived virus, the Egyptian 1977 virus strain ZH501. Although no severe pathology was reported in the ZH501-infected sheep, viremia and febrile response allowed for the evaluation of vaccine efficacy [30].

We recently adopted this model to evaluate the pathogenicity of two genetically-distinct RVFV strains in sheep [31]. The first strain, Saudi Arabia 2001 (SA01), was originally isolated from *Aedes vexans arabiensis* during the 2000–2001 outbreak in the Kingdom of Saudi Arabia [32]. This outbreak had higher human case fatality rates than previously noted (13.9%–33.9%) [33]. The second strain, Kenya 2006 (Ken06), was isolated from *Aedes ochraceus* from Kenya in 2006 [34]. While Ken06 was found to be closely related to viruses isolated in 1991 in Madagascar and in 1997 in Kenya [32], it is genetically distinct from these viruses [31]. Our sheep challenge model for SA01 and Ken06 RVFV demonstrated differences in viremia, microscopic and macroscopic pathological changes and aberrations in hematology and liver enzyme chemistry values. The aim of the present study was to further compare and confirm these virulence differences by evaluating SA01 and Ken06 infections in another important target species, cattle.

## 2. Materials and Methods

### 2.1. Virus Strains and Cell Culture

The RVFV Saudi Arabia 2000–2001 (SA01) [34] and Kenya 2006–2007 (Ken06) isolates [32] were provided by Barry Miller, Centers for Disease Control, Fort Collins, CO, through Richard Bowen, Colorado State University. The two virus strains were passaged once in a C6/36 *Aedes albopictus* cell line (ATCC, Manassas, VA, USA) at 30 °C with MEM culture medium (Life Technologies, Grand Island, NY, USA) supplemented with 10% fetal bovine serum (FBS; Sigma-Aldrich, St. Louis, MO, USA) and 1× antibiotic-antimycotics (Penicillin/Streptomycin/Amphotericin B (PSF); Gibco, Watham, MA, USA). Plaque assays were performed using the Vero MARU (Middle America Research Unit, Panama) cell line grown in M199E culture medium (Sigma-Aldrich, St. Louis, MO, USA) supplemented with 10% FBS and 1× PSF and maintained in a 37 °C, 5% CO_2_ incubator.

### 2.2. Animals and Experimental Design

Twelve healthy Hereford cross or Angus cross cattle, 4–5 months old, were obtained from private breeders in Kansas, USA. The animals were acclimated for one week at the Large Animal Research Center (Kansas State University (KSU), Manhattan, KS, USA) prior to relocation to a Biosafety Level-3 Agriculture (BSL-3Ag) facility at the KSU Biosecurity Research Institute for the virus inoculation experiment. In the BSL-3Ag facility, the animals were divided into two experimental groups of five each. Two additional animals were included as mock-inoculated controls. Cattle in each experimental group were inoculated subcutaneously with 2 mL of 1 × 10^6^ plaque-forming units (pfu)/mL of the Ken06 or SA01 strain. After RVFV exposure, all animals were monitored daily for temperature changes and clinical signs. Nasal swabs for virological analysis and blood samples for virological, immunological, and blood chemistry analyses were collected daily from Days 0–10 and additionally at 14 and 21 days post-infection (dpi). One animal per experimental infection group was euthanized and necropsied at 3, 4, 5, 10 and 21 dpi. Animals were randomly pre-selected for necropsy; however, severe clinical illness required one animal to be substituted earlier in the schedule. The two mock-inoculated control cattle were necropsied at 20 dpi in order to allow for complete and thorough necropsies on all animals under BSL-3Ag conditions. Tissues were collected for viral titer determinations and histopathology. The research was performed under an Institutional Animal Care and Use Committee-approved protocol of KSU in compliance with the Animal Welfare Act and other regulations relating to animals and experiments involving animals.

### 2.3. Virus Isolation and Plaque Titration

Tissue samples of brain, kidney, liver and spleen were collected at necropsy and frozen at −80 °C. Approximately 10 mg of tissue were added to 1 mL M199E supplemented with 10% FBS and 1× PSF and homogenized by high-speed shaking dissociation with steel beads using the TissueLyser instrument (QIAGEN Inc.; Valencia, CA, USA). Virus stocks, cattle sera, nasal swabs and homogenized tissue samples were titered by standard plaque assay on Vero MARU cells, as previously described [35]. Briefly, confluent cell monolayers were inoculated with ten-fold serial diluted samples in M199E and incubated for 1 h. Following adsorption, the inocula were replaced with a 1:1 mixture of 2% carboxymethyl cellulose (Sigma-Aldrich, St. Louis, MO, USA) in 2× M199E (2% FBS and 2× PSF) and returned to the incubator. After five days, cells were fixed and stained with crystal violet fixative (25% formaldehyde, 10% ethanol, 5% acetic acid, 1% crystal violet).

### 2.4. Viral RNA Extraction and Real-Time RT-PCR

Total RNA from serum, nasal swabs or homogenized tissue samples was extracted using TRIzol-LS reagent (Life Technologies, Grand Island, NY, USA) and the magnetic-bead capture MagMAX-96 total RNA Isolation kit (Life Technologies). Briefly, 100 μL of aqueous phase were added to 90 μL of isopropanol and 10 μL bead mix (Beckman Coulter, Danvers, MA, USA). Total sample RNA was washed four times with wash buffer (150 μL) and then eluted in 30 μL of elution buffer. A quadruplex real-time reverse transcriptase-polymerase chain reaction (RT-PCR) assay was used to detect each of the three RVFV RNA genome segments and an external RNA control [36].

#### 2.4.1. RNA Copy Number Determination

*In vitro* transcribed RNA (IVT RNA) was generated using the T7 transcription kit (MEGAscript, ThermoFisher) from a PCR-generated amplicon derived from a DNA plasmid (pBluescript III) using cDNA SuperMix (Quanta Biosciences) and T7 promoter and terminator primers (Integrated Technologies). The RVFV L plasmid (provided by Hana Weingartl, National Centre for Foreign Animal Diseases, Canadian Food Inspection Agency, Manitoba, Canada) contains 3482 base pairs of the L segment of RVFV (nucleotides 1–3482 of the ZH501 RVFV strain). IVT RNA was DNAse treated 3×, column purified (MEGAclear, ThermoFisher) and quantitated with spectrophotometry. The copy number was calculated using an online calculator [37]. Ten-fold serial dilutions of IVT stock RNA (10^4^–10^−1^ copies) were utilized to generate a six-point standard curve using six PCR well replicates per dilution using quantitative RVFV real-time RT-PCR [36]. Copy numbers for samples were mathematically determined using the PCR-determined mean Ct for the L segment (three PCR well replicates) and the slope and intercept of the L segment IVT RNA standard curve. Data are reported as PCR-determined copy number per reaction. Calculated copy numbers less than 15 (equivalent to Ct greater than 35) are considered past the limits of detection for this assay, are classified as equivocal and, thus, are not reported as true positives.

### 2.5. RVFV Serology

#### 2.5.1. Anti-RVFV IgG Antibody Response

The serum was inactivated prior to serological testing by adjusting to 0.25% Tween and incubating at 60 °C for 2 h. Each sample was safety tested by 3 blind passes in cell culture. Only samples that demonstrated no cytopathic effect on the third passage were removed for serology. Anti-RVFV antibody response was measured by an anti-RVFV total IgG indirect enzyme linked immunosorbent assay (ELISA) using recombinant baculovirus-expressed RVFV Gn and N proteins as diagnostic antigens. Briefly, each plate was coated overnight at 4 °C with approximately 150 ng of each purified recombinant protein/antigen, and the ELISA was performed as previously described [38]. The cut-off point for the specific ELISAs was determined by the addition of three standard deviations to the corresponding mean OD value of the pre-vaccination for all of the animals and the control serum. Mean OD values equal to or greater than the cut-off value were considered positive.

#### 2.5.2. Plaque Reduction Neutralization Test

To assess the anti-RVFV neutralizing antibody response to RVFV inoculation, a plaque reduction neutralization test was performed as previously described [39]. Briefly, the stock of MP12 RVFV was diluted to 50 pfu in 250 µL of 1× MEM containing 4% bovine serum albumin (BSA; Sigma-Aldrich). Separately, aliquots of serum from each animal were serially diluted from 1:10–1:1280 in 1× MEM containing 2% BSA and 1% penicillin-streptomycin (Gibco). Diluted serum (250 µL) was mixed with an equal volume of diluted MP12 virus and incubated at 37 °C for 1 h. Thereafter, each mixture of serum plus RVFV was used to infect confluent monolayers of Vero E6 cells (ATCC, Manassas, VA, USA) in 12-well plates. After 1 h of adsorption at 37 °C and 5% CO_2_, the mixture was removed, and 1.5 mL of nutrient agarose overlay (1× MEM, 4% FBS and 0.9% SeaPlaque agar (Lonza Rockland Inc., Rockland, ME, USA)) were added to the monolayers. After 4 days of incubation, the cells were fixed with 10% neutral buffered formalin for 3 h prior to removal of the agarose overlay. The monolayer was stained with 0.5% crystal violet in PBS, and plaques were enumerated. The calculated plaque reduction neutralization test (PRNT_80_) corresponded to the reciprocal titer of the highest serum dilution, which reduced the number of plaques by 80% or more relative to the virus control. As the positive neutralizing serum control, a 1:40 dilution of Day 28 neutralizing serum (titer >1280) obtained from sheep previously immunized with the RVFV glycoprotein subunit vaccine was used [39].

### 2.6. Blood Chemistry Analyses

An aliquot of serum was frozen immediately upon processing for later comprehensive large animal diagnostic panel (ALB, ALP, BUN, CA, CK, GGT, GLOB, MG, PHOS and TP) analyses with a Vetscan VS2 instrument (Abaxis, Union City, CA, USA) according to the manufacturer’s instructions. Vendor-provided normal ranges for cattle were used as reference values.

### 2.7. Liver Pathology

Cattle liver samples were collected at necropsy and placed in 10% neutral buffered formalin for at least 7 days. Liver tissue was trimmed, processed and paraffin-embedded. All histochemical stains and immunohistochemistry were done on 4-µm sections placed on positively-charged slides. Hematoxylin and eosin (H&E)-stained tissues were reviewed by a veterinary pathologist in a blinded fashion and the liver pathology scored per slide (2–3 slides, minimum 3 tissues sections per animal) for lesion severity on a semi-quantitative scale from 0–4, where 0 signified no lesions and 1–4 progressively more severe pathology with a greater degree of liver parenchyma involvement (Table 1). Immunohistochemistry (IHC) for RVFV antigen using the polyclonal rabbit anti-RVFV nucleocapsid protein antibody [40] and an avidin-biotinylated peroxidase complex (ABC) detection technique was conducted on all liver sections as described [31], with the addition of a chromogen-enhancing step. Briefly, slides were deparaffinized and rehydrated, the antigen retrieved using a vegetable steamer technique in pH 6.0 citrate buffer with detergents (DAKO, Carpinteria, CA, USA) for 20 min, blocked with 3% hydrogen peroxide for 10 min, serum blocked as per the kit, incubated overnight at 4 °C with a 1:1000 dilution of primary antibody, secondary antibody, and ABC reagent was applied as per kit, with 3,3’-Diaminobenzidine (DAB) followed by DAB enhancing solution applied as per the vendor instructions (Vector Labs), counterstained with hematoxylin and mounted in Permount (Electron Microscopy Systems, Hatfield, PA, USA). Throughout, the following controls were employed: reagent control slides, with and without equivalent concentrations of primary antibody matched animal serum, and uninfected control cattle liver. Additionally, a Hall’s histochemical stain for bilirubin was run on select liver sections. All gross tissue images were captured with a Canon G12 camera (Cannon, USA Inc., Melville, NY, USA), and microscopic images were captured with a DP25 camera (Olympus, Tokyo, Japan) on a BX46 light microscope (Olympus) using CellSens Standard Version 1.12 (Olympus). All microscopic images were further color calibrated using ChromaCal software ver 2.5 (Datacolor Inc., Lawrenceville, NJ, USA) as per the manufacturer’s instructions and published recommendations (Linden, Sedgewick and Ericson, 2015); the figure panels were composed in Adobe Photoshop and InDesign CC (Adobe, San Jose, CA, USA).

### 2.8. Statistical Analysis

Differences in values of key experimental parameters were analyzed statistically. The values were analyzed using the *t*-test for independent samples. Mean and standard deviations were calculated for animals available per days post-infection (dpi). The small number of animals used due to space constraints (BSL-3Ag) reduced the value of statistical analysis.

## 3. Results

### 3.1. Rectal Temperatures

Rectal temperatures of calves inoculated with SA01 or Ken06 were monitored from 0–10, 14 and 21 dpi (Table 2). Two out of the five animals inoculated with SA01 had a fever (>40 °C) at 2 dpi, while the others were normal throughout the study. At 2 dpi or later, four out of five animals inoculated with Ken06 had a fever for at least one day, and one had a consistent fever for three days (Table 2). Rectal temperatures were within normal limits for all of the animals remaining past 7 dpi.

### 3.2. RT-RCR and Viremia

**Figure 1:**
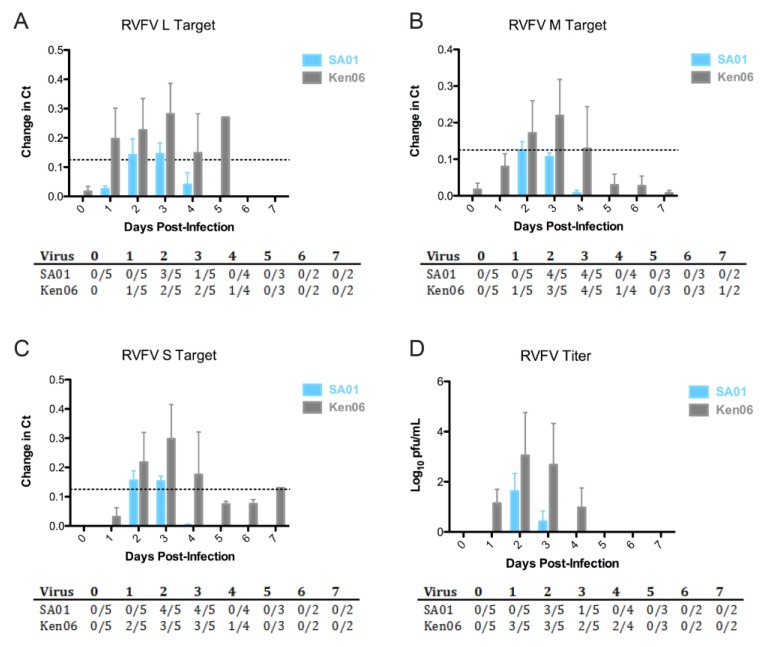
Kinetics of viral RNA and virus titers for calves infected with RVFV strains, SA01 (blue) and Ken06 (gray). The mean with the standard deviation of change in Ct values; (40-Ct)/40 (**A**) RVFV L RNA segment (dashed line indicates standard Ct = 35 cut-off or 0.13 change in Ct), (**B**) RVFV M RNA segment and (**C**) RVFV S RNA segment from calf serum are shown; (**D**) viral titers in pfu/mL. To be considered positive by the multiplex real-time RT-PCR, the Ct value for at least two of the three RVFV genome segments must be less than or equal to 35 [36].

**Figure 2:**
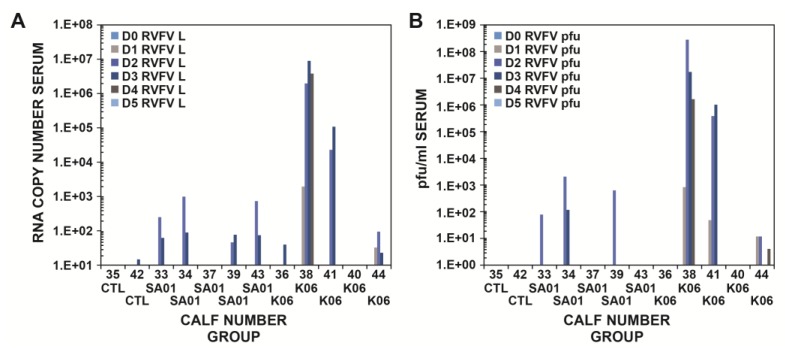
Viral replication dynamics in individual calf serum as determined by molecular and traditional virological methods. (**A**) Quantitative real-time RT-PCR-determined RVFV L segment copy number per reaction calculated from the mean Ct for the RVFV L segment from Day 0–Day 5 post-infection; (**B**) RVF virus titer determined from the plaque assay reported as pfu/mL of serum. PCR Ct equal to or less than 35 equates to greater than 27 copies, which is considered positive and within the quantitative range for quantitative RT-PCR. The X-axis includes calf numbers and group designations; CTL = mock controls, SA01 = infected with RVFV Saudi Arabia 2000–2001; K06 is Ken06 = infected with RVFV Kenya 2006–2007. Calves 43 and 41 were necropsied at dpi 3 and, thus, are not included in the datasets for dpi 4 or 5. Calves 37 and 38 were necropsied on dpi 4 and, thus, are not included in datasets for dpi 5.

Viremia was determined in the cattle sera by both real-time RT-PCR and virus titration up to 21 dpi. Virus was detectable in the serum starting at 1 dpi by both real-time RT-PCR (Figure 1A–C) and the plaque assay (Figure 1D) for the Ken06 group. Viral RNA and virus were detected from the sera of Animal #38 of the Ken06 group at 1 dpi. By 2 dpi, four of five in the SA01 group and three of five in the Ken06 group were positive by real-time RT-PCR, and also, three of five in both groups were viremic (Figure 1A–D). All infected animals were at least weakly positive by real-time RT-PCR (cycle threshold (Ct) ≤37; the standard cut-off threshold is 35), but only three of five animals per group had detectable viremia by virus titration. There was individual animal variation in the detection of viral RNA and virus isolation (Figure 2). The detection of virus occurred just prior to the appearance of fever, at 1 dpi for the Ken06 group, but there was viral RNA and viremia in the SA01 group in the absence of fever. One calf in the Ken06 group had a late fever at 5 dpi and was never positive for viral RNA using the quantitative RT-PCR test criteria Ct ≤ 35 for two of three RVFV gene segments or virus isolation. This animal did have some questionable positive quantitative RT-PCR results (Ct = 35–39) from 5–7 dpi and did have a viral RNA positive nasal swab at 5 dpi.

Peak viremia determined by the plaque assay occurred at 2 and 3 dpi (Figure 1D). Cattle infected with the SA01 strain were all negative by both real-time RT-PCR (Figure 1A–C) and the plaque assay by 4 dpi (Figure 1D). Cattle inoculated with the Ken06 strain were negative by real-time RT-PCR (Figure 1A–C) by 5 dpi, but some weakly positive Ct values above the standard threshold cut-off of 35 were detected out to 7 dpi. No virus plaques were detected by Day 5 dpi for Ken06 (Figure 1D). One animal in the Ken06 group was viremic from 1–4 dpi and died immediately prior to necropsy at 4 dpi. This animal had a peak viremia of 3.4 × 10^8^ pfu/mL at 2 dpi with real-time RT-PCR Ct values of 14–19 depending on the viral segment used for detection. Control co-housed cattle sera remained negative by RT-PCR and virus titration throughout the study.

### 3.3. Viral Load or Titers in Tissues

Brain, kidney, liver and spleen samples collected at necropsy were also tested for virus presence (Table 3). The Ken06 group Animals #41 at 3 dpi and #38 at 4 dpi had viral RNA-positive brain tissue (average of L, M and S Ct = 34 and 31, respectively). Viral RNA was also found in the kidneys of the Ken06 group cattle, #41 at 3 dpi (average of L, M and S Ct = 28) and #38 at 4 dpi (average of L, M and S Ct = 23), as well as the SA01 group Animal #34 at 5 dpi (average of L, M and S Ct = 26). Virus was isolated from the kidneys of #38 (1.4 × 10^5^ pfu/mL) and #34 (1.5x10^2^ pfu/mL). Liver samples from both groups were positive for viral RNA from 3–4 dpi and at 5 dpi for the SA01 group (Ct = 18–30). At 5 dpi, the SA01 animal’s liver tissue was positive for viral RNA (Ct = 29), but the Ken06 was inconclusive (Ct = 38). Virus was isolated from the liver of both groups at 3 dpi and for a Ken06 animal at 4 dpi (Table 3). The spleen results reflected the liver results for viral RNA detection with Ct values from 21–33 from 3–5 dpi. Virus was isolated only from the spleens of Ken06 animals at 3 and 4 dpi with titers of 1.2 × 10^3^ pfu/mL and 2.9 × 10^5^ pfu/mL, respectively. The spleens were positive for viral RNA at 10 dpi from both groups (SA01, Ct = 33; Ken06, Ct = 35). The spleens from both groups were weakly positive at 21 dpi (Ken06, Ct = 36) and inconclusive (SA01, Ct = 38). Nasal swabs from both groups were sporadically positive for viral RNA from 3–6 dpi (total of nine positive samples; Ct = 26–34; data not shown). One swab from Animal #38 was found virus positive (32 pfu/mL). The greatest number of positive nasal swabs was at 3 dpi with three out of five for the SA01 group (average of L, M and S Ct = 32) and two out of five for the Ken06 group (average of L, M and S Ct = 34). Virus was isolated from the nasal swab of only the Ken06 group Calf #38 that also had the highest serum virus titer of 3.5 × 10^8^ pfu/mL, whereas the highest titer of the SA01 group found in Calf #34 was 2.2 × 10^3^ pfu/mL.

### 3.4. Serological Responses

**Figure 3:**
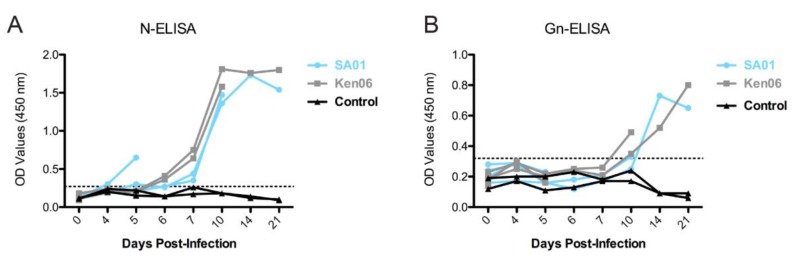
Development of the antibody response of individual calves infected with RVFV strains, SA01 (blue) and Ken06 (gray). Specific indirect ELISA shows the kinetics of total IgG antibody responses in sheep inoculated with wild-type RVFV strains, SA01 and Ken06: (**A**) RVFV N-ELISA; (**B**) RVFV Gn-ELISA. The dashed line indicates the calculated cut-off values (N-ELISA = 0.27; Gn-ELISA = 0.33).

Calves showed the first indication of seroconversion at 6 dpi with OD values above the cut-off points for reactivity to N and 10–14 dpi for reactivity to Gn (Figure 3A, B). Serum samples from 1–3 dpi were not included in the analysis because our standard inactivation procedure failed to fully inactivate the virus due to the presence of high virus titers in some of the sera. Neutralizing antibody responses were detected in remaining animals at 5 dpi and increased until peaking at 10–14 dpi (Table 4) consistent with the ELISA data and indicating serological conversion in response to the experimental RVFV inoculations. None of the co-housed control cattle seroconverted.

### 3.5. Pathology

**Figure 4:**
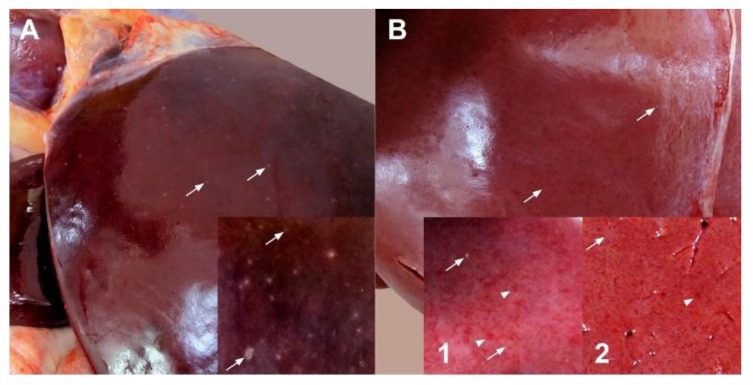
Gross pathology of acute post-infection, time-point livers from virus-inoculated cattle. (**A**) This liver from SA01-inoculated, 3 dpi, Animal #43 shows the typical acute time-point hepatic pathology seen in the 3–5 dpi animals regardless of virus inoculum. Disseminated throughout the parenchyma are myriads of 1–2-mm tan foci (white arrows), necrosis. (**B**) This liver from severely affected Ken06-inoculated, 4 dpi, Animal #38 is diffusely pale. Disseminated throughout the parenchyma are multifocal to coalescing foci of necrosis (white arrows) and hemorrhage (white arrowheads). Inset 1 is a capsular liver view similar to the inset in (A), while Inset 2 shows a cross-section of hepatic parenchyma.

In general, gross pathology observations at 3–5 dpi revealed no consistent pattern of difference between the SA01 and Ken06 virus strains groups; all had 1–3-mm multifocal tan foci (necrotic foci) disseminated throughout their hepatic parenchyma (Figure 4A). However, gross findings for Animal #38 (4 dpi Ken06 inoculated) were more severe. This animal had multifocal to coalescing hepatic necrosis accompanied by marked, multifocal hemorrhage disseminated throughout its hepatic parenchyma (Figure 4B). Additionally, #38 had clinical signs and lesions suggestive of disseminated intravascular coagulation, including hemorrhage and edema in multiple viscera, many edematous and congested lymph nodes throughout its body, renal pelvic hemorrhage, splenic ecchymoses, marked and diffuse pulmonary congestion, multifocal endocardial hemorrhage and red urine observed ante-mortem. In all animals necropsied at 10 dpi and later, gross changes in hepatic parenchyma were no longer evident.

**Figure 5:**
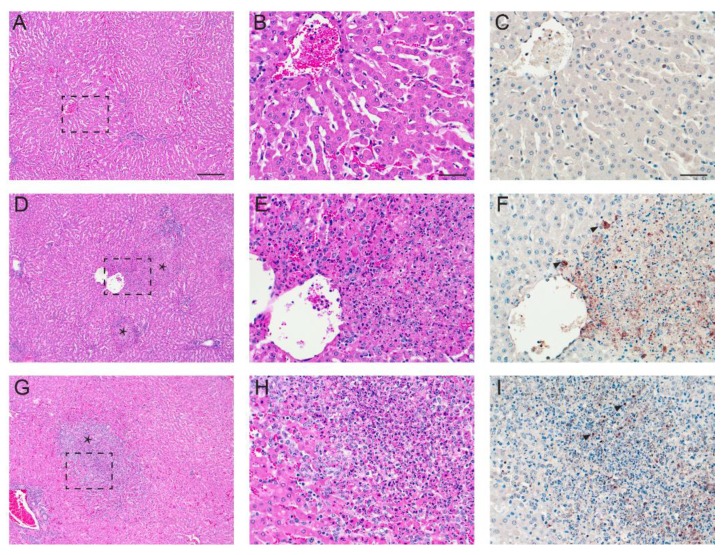
Acute time-point liver histopathology and viral antigen immunohistochemistry. (**A**) Shown is a low magnification hematoxylin and eosin (H&E) stain of the normal liver histology seen in mock-inoculated Animal #42; the black broken line box outlines the area magnified in (**B**); (**C**) viral antigen immunohistochemistry is negative for RVFV antigen; (**D**) the depicted Ken06, 3 dpi, Animal #41 liver H&E is representative of the acute time-point histopathology seen in animals inoculated by this virus; the black stars mark necrotic foci; a portion of one focus is magnified in **(E)**; **(F**) shown is the same necrotic focus, which is strongly positive for RVFV antigen in hepatocytes, macrophages and cellular debris; black arrowheads indicate cytoplasmic positive labeling; (**G**) the depicted SA01, 3 dpi, Animal #43 liver H&E is typical of acute time-point liver histopathology for this virus strain; the black star denotes a single large necrotic focus further magnified in (**H**); acute time-point SA01 liver foci labeled sporadically and less strongly for RVFV antigen when compared to Ken06 foci; (**I**) a stronger than average viral antigen labeling for this larger than average necrotic focus in Animal #43; black arrowheads denote RVFV antigen labeling. Column 1 images are 100× magnification. The bar is 20 μm. Columns 2 and 3 images are 400× magnification. Bars are 50 μm.

Similar to the gross lesion results, we did not find RVFV strain-dependent patterns between the two groups in terms of hepatic histopathology changes. RVFV-infected cattle had severe hepatic lesions at 3–5 dpi, averaging no less than 2.5 (scale 0–4) for their hepatic histopathology score (Table 5 and Figure 5). All of these animals had multifocal mid-zonal to central hepatocellular necrosis disseminated throughout their hepatic parenchyma accompanied by predominantly lymphohistiocytic, inflammation and increased numbers of degenerate and viable neutrophils in larger necrotic foci. The lesions tapered off by 10 dpi (score ≤2) and by 21 dpi were characterized by occasional foci of inflammation, predominantly aggregates of lymphocytes and macrophages.

In contrast, while the sample size per time-point prevented us from appreciating histopathologic differences attributable to virus strain, we saw a consistent difference in immunohistochemical (IHC) labeling for RVFV nucleoprotein antigen in the hepatic lesions at 3 and 4 dpi SA01 cattle. As seen in our prior challenge model work in sheep [31], SA01 hepatic lesions contained notably less viral antigen signal than hepatic lesions in time-point matched Ken06 cattle. Figure 5F shows a typical Ken06 hepatic lesion IHC signal (Figure 5F), whereas Figure 5I shows a stronger than average labeling of a hepatic lesion in an SA01 animal (Figure 5I). Finally, all examined cattle livers, regardless of inoculum type, had mild, peri-portal lymphoplasmacytic inflammation, interpreted as background inflammation unrelated to the study. Additionally, mock-inoculated, co-housed Animal #42, unlike its mock-inoculated peer, Animal #35, had low numbers of scattered foci of mixed inflammation in central and mid-zonal areas. These foci were negative for RVFV antigen and may be attributable to an unrelated etiology.

### 3.6. Blood Chemistry

Blood chemistries were run on all sera available from 0–7 dpi. Of the 14 parameters assessed, significant elevations were seen in multiple hepatic enzymes, BUN, an indicator of renal damage, as well as creatinine kinase (CK), an indicator of skeletal muscle damage (data not shown). These changes were all found in RVFV-infected animals with the exception of CK. Aspartate amino transferase (AST), the hepatic leakage enzyme consistent with acute liver damage, was elevated above normal range (66–211 U/L) in two of the Ken06 group animals, #38 (2–4 dpi; 279, 1959, 1742 U/L, respectively) and #41 (3 dpi; 452 U/L). Using ALP values normalized to 0 dpi values for individual animals, the only animal with a significant elevation in its ALP was Ken06 Animal #38 starting at 3 dpi (0–4 dpi; 149, 151, 155, 451, 476 U/L, respectively). Animal #38 was also the only one with an elevation in gamma-glutamyl transferase (GGT) at 3–4 dpi (90 and 100 U/L, respectively). This animal had the most severe hepatic histopathology as documented above, but no evidence of cholestasis (associated with high ALP and GGT values) was seen during the histopathology analysis. An additional check with a Hall’s bilirubin stain on multiple liver sections confirmed an absence of bilirubin in bile canaliculi, a hallmark of cholestasis. Animal #38 also had high BUN levels at 4 dpi (32 mg/dL), immediately prior to death. Finally, Animal #38 and SA01 group Animal #37 both had a single elevated CK value accompanied by a high normal value on a consecutive day indicating muscle damage, #37 (3–4 dpi; 797 and 504 U/L, respectively) and #38 (3–4 dpi; 585 and 834, respectively). Interestingly, while Animal #38 had severe clinical signs, Animal #37 did not show obvious clinical signs; it did have significant hepatic pathology (see the pathology section). One of the mock-inoculated, co-housed cattle had a single elevated CK value (1175 U/L), most likely due to difficult restraining procedures during sampling.

## 4. Discussion

The development of reliable challenge models for arboviral disease is often challenging because needle inoculation does not mimic natural infection via insect vectors. There has been significant effort made in the development of vaccines for RVF [23,26,41,42,43]; thus, it is important that reliable animal models using a variety of challenge viruses are available for vaccine evaluation in all target species. For example, previous RVFV challenge models have used the 1977 strain from Egypt, ZH501, in goats and sheep [29]. While this model does provide a viremia and fever response in sheep sufficient to evaluate vaccines [30], it does not produce clinical disease. In a previous study using two more recently-isolated, genetically-distinct strains of RVFV (SA01 and Ken06) and sheep (Dorper/Katahdin cross and Polypay), we observed clinical disease [31]. In this sheep study, we also found that the Ken06 produced greater liver pathology based on blood chemistry markers and histopathology. This finding was surprising because preliminary studies that compared ZH501 with another RVFV isolate from Kenya did not demonstrate any differences in calf responses to infection (Weingartl *et al.*, unpublished data). Therefore, the current study was performed to confirm and determine if the differences in clinical signs after infection with genetically-distinct RVFV strains noted in sheep would also be found in cattle.

In the previous sheep studies, the febrile response was found to be consistent across all RVFV-infected animals. In this calf study, the febrile response was less consistent with both isolates. Only two out of five SA01-inoculated calves developed a mild fever and only for one day. In the Ken06 group, four out of five had a fever for one day, and one animal was febrile for three days (Table 2). In most cases, the fever occurred at 2 dpi when viremia was beginning to peak. Viral RNA was detectable in the liver and spleen for both virus groups at 3 dpi and in the brain and kidneys of Ken06 group animals at 3 and 4 dpi. The spleens of both virus groups had detectable viral RNA starting at 3 dpi up to 21 dpi. Virus was detected at low titer in SA01 animals in the liver and kidney at 3 and 5 dpi, respectively. Virus was isolated with titers from 10^3^–10^6^ pfu/mL from the liver and spleen at 3 and 4 dpi and from the kidney at 4 dpi for the Ken06 animals. Although viral RNA was detected in all four tissues examined (brain, kidney, liver and spleen), the liver and spleen were the most consistently positive by both RT-PCR and virus isolation. The presence of viral RNA in the spleen post-viremia potentially out to 20 dpi suggests this may be a good target tissue for diagnosis. There was considerably more variation in the calf responses (Figure 2) than seen in the sheep study [31]. This may have been in part because of the use of mixed breed calves due to their availability at the time of the study. No breed susceptibility conclusions can be drawn due to the small number of animals used.

Nasal swabs from a few animals of both groups were sporadically positive for viral RNA. Ken06 Animal #38 with the highest viremia was the only animal that also had a virus-positive nasal swab (32 pfu/mL). Mock-inoculated control calves co-housed with the challenged animals remained negative for all RVFV-specific parameters throughout the study, suggesting that RVFV was not shed at levels necessary for transmission among cattle. This is consistent with the view that RVFV is most often transmitted through an infected mosquito bite. However, although the potential for transmission through nasal discharge is low, it should not be entirely ruled out.

The blood chemistry analysis indicated primarily liver involvement in most affected animals. As with the previous study in sheep [31], AST appears to be the most consistent marker of clinical disease. The small number of animals in the present study and the removal of animals for necropsy at 3, 4 and 5 dpi during the peak of viremia could have affected these results; however, the liver function results are consistent with a previous experimental infection of calves using seven- and 21-day-old animals [44]. Because the renal function marker BUN was also elevated in the most severely affected animal, kidney function may also be worth monitoring in future studies.

Regardless of virus strain, at the acute time-point (3–5 dpi) post-infection, calf livers had grossly visible multifocal necrosis that in the most severe case, Animal #38, was accompanied by marked hemorrhage (Figure 4 and Figure 5). These lesions contained RVFV antigen-positive cells. Differences in hepatic histopathology due to virus strain were not noted. This could be due to the study design of one animal per virus, per time-point. However, similar to what was seen in our sheep study [31], the comparison of hepatic lesions from acute time-point animals for both viruses revealed that Ken06 lesions on average labeled more strongly positive for RVFV antigen (Figure 5). Lesions found in this study were consistent with those reported in previous cattle studies [28,44,45].

Most recent studies of RVFV pathogenesis and vaccine evaluation focus on more susceptible target species, such as sheep. This is the first study to examine virus replication and pathological development of two distinct RVFV strains during the clinical stage of infection in cattle. Although the fever and viremia were less consistent in cattle than sheep, the vaccine-age calf model did display sufficient clinical responses to be useful for efficacy evaluation of cattle vaccines against RVF.

## Figures and Tables

**Table 1:** Liver histopathology score descriptions.

Histopathology Score	Description
0	Multifocal, peri-portal, mild lymphoplasmacytic (lymphocytes and plasma cells) inflammation (background lesion)
1	Multifocal, mid-zonal to central foci of lymphohistiocytic (lymphocytes and macrophages) inflammation with lesser numbers of plasma cells and occasional single hepatocyte necrosis accompanied by low numbers of neutrophils
2	Multifocal, up to 1-mm areas of mid-zonal to central lymphohistiocytic inflammation involving up to 5% of the examined parenchyma; in the foci with central necrosis, the inflammation shifts to predominantly neutrophils; less than 5% of examined parenchyma involved
3	As prior, but including scattered necrotic foci that have >1 mm-diameter areas and involving up to 20% of the hepatic tissue reviewed; scattered hepatocyte apoptosis is additionally present
4	Greater than 20% of the hepatic parenchyma involved with lesions, as described previously; additionally, there is prominent multifocal hemorrhage

**Table 2:** Kinetics of the rectal temperature (°C) of calves infected with Rift Valley fever virus (RVFV) strains, Saudi Arabia 2001 (SA01) and Kenya 2006 (Ken06) (red indicates above normal for cattle, >40 °C).

		Days Post-Infection							
Strain	No.	0	1	2	3	4	5	6	7
SA01	33	39.4	38.7	41.3	38.8	39.0	38.6	39.8	38.3
	34	39.2	38.8	39.6	38.9	38.7	38.5		
	37	38.9	38.9	38.8	39.0	38.7			
	39	39.1	38.4	40.9	39.8	39.2	38.8	38.9	38.6
	43	39.8	38.9	39.5					
Ken06	36	40.4	39.4	39.3	38.3	38.7	38.8		
	38	38.9	38.9	41.5	41.6	41.1			
	40	39.0	38.8	38.8	38.9	38.8	40.8	39.3	38.5
	41	39.2	39.1	40.8	39.8				
	44	39.4	39.7	40.7	39.5	39.1	38.3	38.3	38.5
Control	35	39.1	39.4	38.8	38.6	38.4	38.7	38.3	38.3
	42	39.0	40.3	38.5	38.9	39.3	38.6	38.8	38.5

**Table 3:** Presence of RVFV RNA and virus in tissues at days post-infection.

		Days Post-Infection									
		**3**		**4**		**5**		**10**		**20**	
		*Ct*	*Titer*	*Ct*	*Titer*	*Ct*	*Titer*	*Ct*	*Titer*	*Ct*	*Titer*
*SA01*	Brain	ND	-	ND	-	ND	-	ND	-	ND	-
	Kidney	ND	-	37	-	26	1.5 × 10^2^	ND	-	ND	-
	Liver	30	4.0 × 10°	30	-	29	-	37	-	ND	-
	Spleen	31	-	33	-	32	-	33	-	38	-
*Ken06*	Brain	34	-	31	-	38	-	ND	-	ND	-
	Kidney	28	-	23	1.4 × 10^5^	ND	-	ND	-	ND	-
	Liver	20	2.1 × 10^5^	18	3.9 × 10^6^	38	-	ND	-	ND	-
	Spleen	24	1.2 × 10^3^	22	2.9 × 10^5^	ND	-	35	-	36	-

Titer: pfu/mL from 10 mg homogenate in 1 mL of media; Ct = cycle threshold mean of S, L, M, real-time RT-PCR; ND = not detected or Ct of 40; - = no plaque formation.

**Table 4:** Reciprocal plaque reduction neutralization test (PRNT_80_) titers in calves infected with RVFV strains, SA01 and Ken06.

		Days Post Infection							
**Strain**	**No.**	**0**	**4**	**5**	**6**	**7**	**10**	**14**	**21**
*SA01*	33	-	-	10	40	160	640	1280	1280
	39	-	-	10	160	320	>1280		
	34	-	-	-					
	37	-	-						
	43	-							
Mean				10	100	240	960		
*Ken06*	40	-	-	40	40	80	1280	640	>1280
	44	-	-	40	80	320	1280		
	36	-	-	-					
	38	-	ND						
	41	-							
Mean				40	60	200	1280		

- = negative; ND = not determined.

**Table 5:** Liver histopathology and immunohistochemistry for RVFV antigen.

Strain	Calf No.	Days PI	H Score	IHC	PCR	Titer
*SA01*	43	3	3	+	+	4.0 × 10°
	37	4	2.5	+	+	-
	34	5	3	+	+	-
	39	10	2	-	-	-
	33	21	1	-	-	-
*Ken06*	41	3	3	+	+	2.1 × 10^5^
	38	4	4	+	+	3.9 × 10^6^
	36	5	1	+ *	-	-
	44	10	2	-	-	-
	40	21	1	-	-	-
Mock	35	20	0	-	-	-
	42	20	0	-	-	-

H Score is the average hepatic histopathology score on a scale from 0, no lesions, to 4, severe lesions (Table 1), for 3–5 reviewed sections of liver per animal. IHC is the anti-RVFV immunohistochemistry (IHC) result on liver sections; a positive “+” is given when at least one section of liver was positive for viral antigen. Key: + = positive for viral antigen by IHC; - = negative for viral antigen by IHC. PCR is the quantitative RT-PCR result for liver tissue. * No viral antigen-positive lesions; a positive cytoplasmic signal is present in low numbers of circulating Kupffer cells (macrophages). The titer is pfu/mL of tissue homogenate from 10 mg of tissue in 1 mL of media (- = no plaques).
